# Comprehensive bioinformatics analysis reveals potential lncRNA biomarkers for overall survival in patients with hepatocellular carcinoma: an on-line individual risk calculator based on TCGA cohort

**DOI:** 10.1186/s12935-019-0890-2

**Published:** 2019-07-04

**Authors:** Zhiqiao Zhang, Yanling Ouyang, Yiyan Huang, Peng Wang, Jing Li, Tingshan He, Qingbo Liu

**Affiliations:** 1 Department of Infectious Diseases, Shunde Hospital, Southern Medical University, Shunde, 528308 Guangdong China; 2 Department of Hepatobiliary Surgery, Shunde Hospital, Southern Medical University, Shunde, 528308 Guangdong China

**Keywords:** Long non-coding RNA, Hepatocellular carcinoma, Overall survival, Prognosis, Nomogram

## Abstract

**Background:**

Accumulated evidences have demonstrated that long non-coding RNAs (lncRNAs) are correlated with prognosis of patients with hepatocellular carcinoma. The current study aimed to develop and validate a prognostic lncRNA signature to improve the prediction of overall survival in hepatocellular carcinoma patients.

**Methods:**

The study cohort involved 348 hepatocellular carcinoma patients with lncRNA expression information and overall survival information. Through gene mining approach, the current study established a prognostic lncRNA signature (named LncRNA risk prediction score) for predicting the overall survival of hepatocellular carcinoma patients.

**Results:**

The current study built a predictive nomogram based on ten prognostic lncRNA predictors through Cox regression analysis. In model group, the Harrell’s concordance indexes of LncRNA risk prediction score were 0.811 (95% CI 0.769–0.853) for 1-year overall survival, 0.814 (95% CI 0.772–0.856) for 3-year overall survival and 0.796 (95% CI 0.754–0.838) for 5-year overall survival respectively. In validation cohort, the Harrell’s concordance indexes of LncRNA risk prediction score were 0.779 (95% CI 0.737–0.821), 0.828 (95% CI 0.786–0.870) and 0.796 (95%CI 0.754–0.838) for 1-year survival, 3-year survival and 5-year survival respectively. LncRNA risk prediction score could stratify hepatocellular carcinoma patients into low risk group and high risk group. Further survival curve analysis demonstrated that the overall survival rate of high risk patients was significantly poorer than that of low risk patients (*P *< 0.001).

**Conclusions:**

In conclusion, the current study developed and validated a prognostic signature to predict the individual mortality risk for hepatocellular carcinoma patients. LncRNA risk prediction score is helpful to identify the patients with high mortality risk and optimize the individualized treatment decision. The web calculator can be used by click the following URL: https://zhangzhiqiao2.shinyapps.io/Smart_cancer_predictive_system_HCC_3/.

**Electronic supplementary material:**

The online version of this article (10.1186/s12935-019-0890-2) contains supplementary material, which is available to authorized users.

## Background

Hepatocellular carcinoma (HCC), as a serious public health problem, is the sixth most common malignant tumor and ranks second in the causes of cancer related death [1]. Since HCC patients at early stage usually had no obvious symptoms, most HCC patients were diagnosed at advanced stage. Despite the great advances in terms of early diagnosis and clinical therapy, the overall survival (OS) of HCC patients remains unsatisfactory [2]. It has been reported that the actual 10-year survival rate was merely 7.2% after surgical resection through a meta analysis with 4197 HCC patients [3]. Therefore, a reliable prognostic signature is needed to monitor HCC patients with poor prognosis and subsequently optimize the clinical treatment decision.

Long non-coding RNAs (lncRNAs), as a class of RNAs > 200 nucleotides in length, may act important roles in biological processes [4, 5]. Several lncRNAs have been reported to be correlated with survival of HCC patients [6, 7]. Recently, several prognostic signatures based on lncRNA expression data have been built to predict the prognosis of HCC patients [8–10]. However, these were several limitations for clinical application of these previous prognostic signatures. Firstly, these prognostic signatures provided only simple scores of overall survival but not percentages of individual mortality risk. Secondly, it is too difficult to calculate the risk scores through these complicated prognostic signatures. Meanwhile, the difference and influence of different gene detection platforms and different transformation methods of original gene expression values should be taken into account for clinical application of these prognostic signatures.

Therefore, the present study aimed to build and validate a prognostic model to predict the prognosis of HCC patients using lncRNA expression data downloaded from The Cancer Genome Atlas (TCGA) database. The present study was carried out in accordance with the suggestions by Transparent Reporting of a multivariable prediction model for Individual Prognosis Or Diagnosis (TRIPOD) [11].

## Materials and methods

### Protocol approval

The present study downloaded the original study dataset from The Cancer Genome Atlas (TCGA) database. The download and analysis of the study dataset strictly adhered to the relevant data policies of TCGA database.

### The gene expression dataset

The gene expression dataset was downloaded from TCGA database (January 28, 2018, https://tcga-data.nci.nih.gov/docs/publications/tcga/). The original gene expression data were generated on illumina HiSeq 2000 RNA Sequencing platform. The download gene expression dataset involved 371 hepatocellular carcinoma samples and 50 normal samples with 60,488 original gene expression values. The lncRNAs descripted in GENCODE Resource database (release 27, mapped to GRCh37, https://www.gencodegenes.org/) were selected for further study. There were 14,449 lncRNAs included in the present study for further analysis.

### Differential expression analysis

The lncRNAs which original expression values < 1 were filtered out from the present study. Then the lncRNA expression values were further standardized through method of Trimmed Mean of M [12]. The criteria of differential gene selection were *P* value < 0.05 and |log_2_fold change| > 2.

### Clinical dataset

**Fig. 1:**
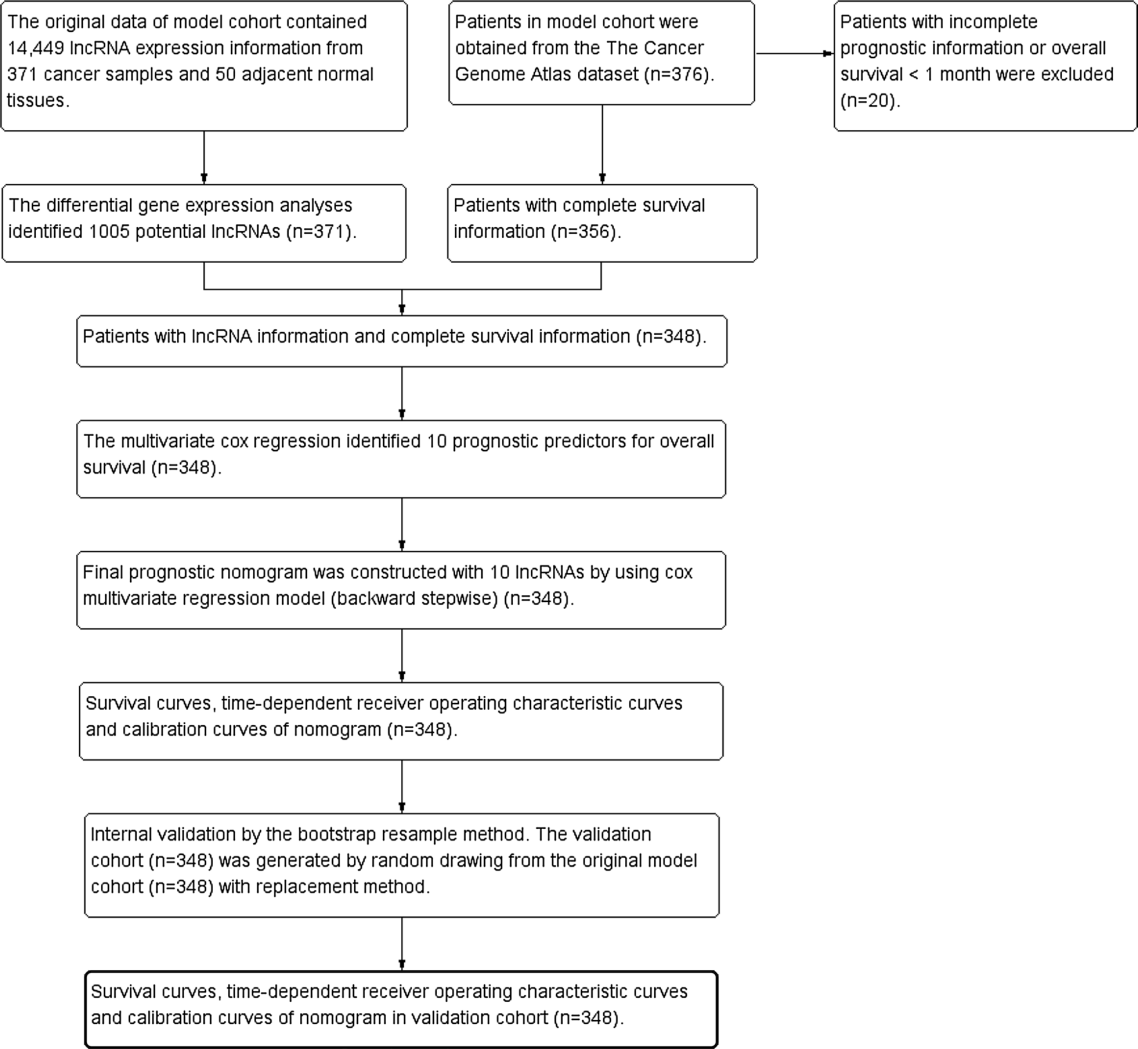
The flowchart in the current study. *TCGA* The Cancer Genome Atlas

There were 376 HCC patients in the clinical dataset from TCGA database. The study endpoint in the current study was overall survival. To avoid the effects of unrelated confounding factors, 20 HCC patients with overall survival less than 1 month were excluded from the present study. Eight patients without lncRNA expression information were excluded from the present study. Finally, there were 348 HCC patients enrolled the final survival analysis (Fig. 1). The study period of The Cancer Genome Atlas Liver Hepatocellular Carcinoma (TCGA-LIHC) cohort was from 2010 to 2015. The maximum value and the minimum value of the overall survival time were 120.7 months and 1.0 month. The missing data were recorded as “NA” in the present study. The mean ± standard deviation of age of HCC patients was 59.5 ± 13.4 years in model group. The mean ± standard deviation of follow-up period was 840 ± 701 days. There were 130 (37.4%) out of 348 HCC patients died in the follow-up period.

### Internal validation

We carried out an internal validation to validate the predictive performance of the present prognostic model. The validation dataset was constructed by drawing 348 HCC patients using bootstrap resampling method, which was recommended for internal validation of prognostic model [13, 14].

### Statistical analysis

Continuous variables in the present study were presented as mean ± standard deviation (SD). The *t*-test or Mann–Whitney U test was performed to compare the differences of continuous variables as appropriate. The Chi-squared test or Fisher’s exact test was performed to compare the differences of categorical variables as appropriate. Time-dependent receiver operating characteristic (ROC) curves and Harrell’s concordance index (C-index) were performed to assess the predictive accuracy of prognostic models. The statistical analyses were carried out by using SPSS Statistics 19.0 (SPSS Inc., an IBM Company) and R software (version 3.4.4). The following R packages, such as “pROC”, “plyr”, “rms”, “survival”, “timeROC “ and “glmnet “, were performed as appropriate in the present study. *P* < 0.05 was defined as the criteria of statistical significance.

## Results

### Study group

Three hundred and forty-eight HCC patients were eventually included in the final survival analysis. The average age of 348 HCC patients was 59.5 ± 13.4 years and the average overall survival time of 348 HCC patients was 28.0 ± 23.7 months in the current study. One hundred and thirty (37.4%) patients out of 348 HCC patients died within the follow-up period in model group. The comparisons of basic characteristics between model group (Additional file 1) and validation cohort (Additional file 2) were summarized in Table 1. There were no significant differences in terms of basic characteristics between model group and validation cohort.

**Table 1:** The clinical features of hepatocellular carcinoma patients in model cohort and validation cohort

	Model group (n = 348)	Validation cohort (n = 348)	*P* value
Death [n (%)]	130 (37.4)	123 (35.3)	0.582
Survival time (mean ± SD, month)	28.0 ± 23.7	26.6 ± 22.6	0.439
Age (mean ± SD, year)	59.5 ± 13.4	59.6 ± 13.4	0.569
Gender (male/female)	236/112	236/112	1.0
AJCC stage (IV/III/II/I/NA)	4/80/79/164/21	7/70/67/182/22	0.226
AJCC PT (T4/T3/T2/T1/NA)	14/74/87/171/2	10/71/75/192/0	0.163
AJCC PN (N3/N2/N1/N0/NA)	100/3/245/0	117/3/228/0	0.165
AJCC PM (MX/M1/M0/NA)	96/4/248/0	103/7/238/0	0.392
Radiation treatment adjuvant (yes/no/NA)	4/130/114	5/225/118	0.631
Pharmaceutical adjuvant (yes/no/NA)	15/215/118	12/213/123	0.592
Ablation embolization (yes/no/NA)	13/219/116	18/210/120	0.328

Continuous variables were compared by using t-test or Mann–Whitney U test as appropriate; categorical variables were compared by using Chi-squared test or Fisher’s exact test as appropriate

*NA* missing data, *SD* standard deviation, *AJCC* American Joint Committee on Cancer

### Differential expression analysis

The differential expression analysis between 371 cancer samples and 50 normal samples was performed by using “edgeR” package. Through “edgeR” package, one thousand and five lncRNAs were identified for further survival analysis. The heat map was presented in Additional file 3: Figure S1 and volcano map was presented in Additional file 4: Figure S2.

### Construction of prognostic nomogram

The univariate Cox regression analyses were conducted to screen the potential lncRNA predictors for overall survival of HCC patients. Based on the potential lncRNA candidates identified by univariate Cox regression analyses, ten lncRNA predictors for overall survival were finally ascertained through multivariate Cox regression analysis. The relevant model information of ten lncRNA candidates were presented in Table 2. The median values of lncRNA expression values were used as cut-off values to transform the original lncRNA expression values into “1” (as high expression) and “0” (as low expression).

**Table 2:** The model information of ten prognostic lncRNA predictors in Cox regression

Variables	Univariate analysis			Multivariate analysis			
	HR	95% CI	*P*-value	Coefficient	HR	95% CI	*P*-value
LINC01559 (high/low)	1.952	1.370–2.780	< 0.001	0.771	2.163	0.480–3.159	< 0.001
MYLK_AS1 (high/low)	1.717	1.206–2.443	0.003	0.528	1.695	1.167–2.462	< 0.001
RP11_150O12.3 (high/low)	1.735	1.223–2.461	0.002	0.728	2.070	1.436–2.984	< 0.001
RP11_92C4.6 (high/low)	0.5826	0.410–0.827	0.003	− 0.509	0.601	0.419–0.865	< 0.001
RASGRF2_AS1 (high/low)	0.6571	0.464–0.931	0.018	− 0.765	0.465	0.317–0.683	< 0.001
LINC01116 (high/low)	1.491	1.053–2.112	0.025	0.731	2.077	1.410–3.059	< 0.001
C2orf48 (high/low)	1.694	1.194–2.404	0.003	0.563	1.756	1.214–2.541	0.003
LINC00856 (high/low)	1.719	1.206–2.449	0.003	0.418	1.519	1.033–2.234	0.034
LINC02003 (high/low)	1.482	1.045–2.10	0.027	0.483	1.620	1.118–2.347	0.011
RP11_363N22.3 (high/low)	1.699	1.197–2.413	0.003	0.432	1.540	1.054–2.250	0.026

The medians of lncRNA expression values were used as cut-off values to stratify lncRNA expression values into high expression group (as value 1) and low expression group (as value 0)

*HR* hazard ratio, *CI* confidence interval

**Fig. 2:**
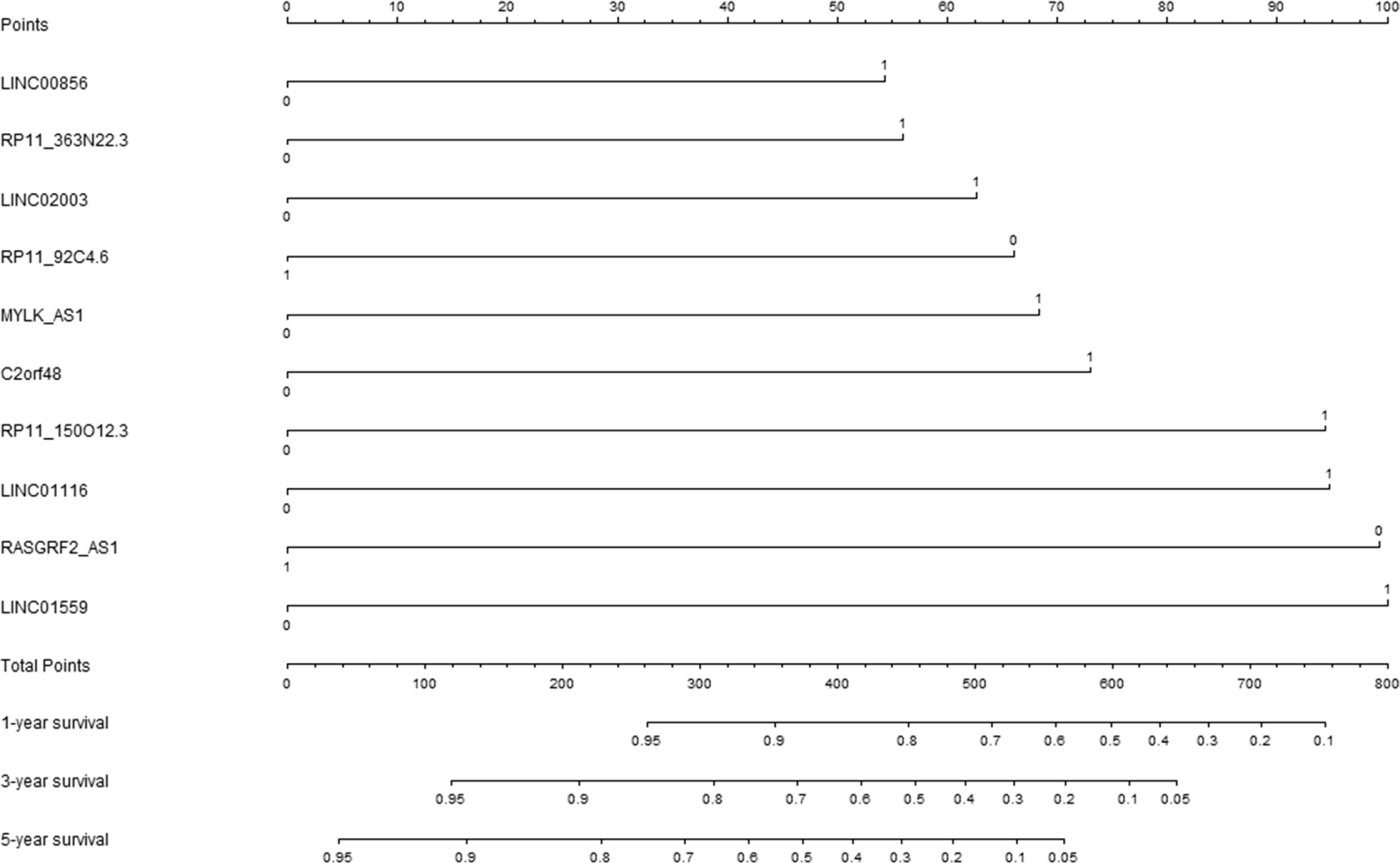
The LncRNA risk prediction score for prediction of overall survival in hepatocellular carcinoma patients

Therefore, a prognostic nomogram (Fig. 2) was built by using the expression values of ten lncRNA predictors: LncRNA risk prediction score = (LINC01559 * 0.771) + (MYLK_AS1 * 0.528) + (RP11_150012.3 * 0.728) − (RP11_92C4.6 * 0.509) − (RASGRF2_AS1 * 0.765) + (LINC01116 * 0.731) + (C2orf48 * 0.563) + (LINC00856 * 0.418) + (LINC02003 * 0.483) + (RP11_363N22.3 * 0.432).

### Predictive performance of LncRNA risk prediction score

**Fig. 3:**
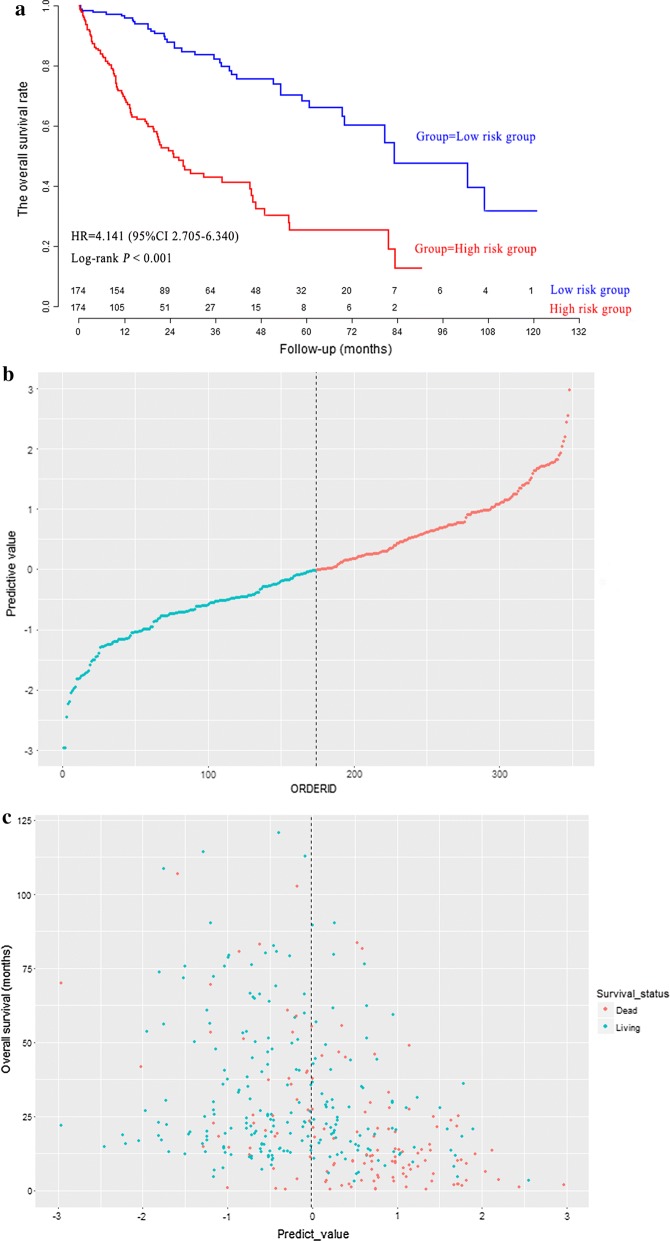
The survival curves of hepatocellular carcinoma patients in model group (**a**). The distribution of LncRNA risk prediction score (**b**), survival status and survival time (**c**) in model group

Through the median value of LncRNA risk prediction score, 348 patients in model group were stratified into low risk group (n = 174) and high risk group (n = 174). As shown in Fig. 3a, the overall survival rate of low risk patients was significantly higher than that of high risk patients (*P *< 0.001). The distribution of LncRNA risk prediction score was presented in Fig. 3b. The overall survival status and overall survival time were presented in Fig. 3c. The Harrell’s concordance index (C-index) of LncRNA risk prediction score was 0.761 (95% CI 0.719–0.803) for overall survival in model group.

### Clinical application of LncRNA risk prediction score

**Fig. 4:**
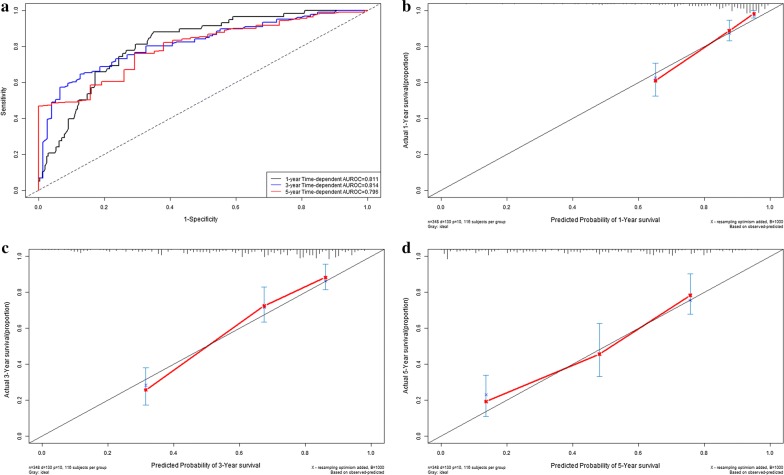
Performance of LncRNA risk prediction score in model group: time-dependent receiver operating characteristic curves (**a**); calibration curve for 1-year overall survival (**b**); calibration curve for 3-year overall survival (**c**); calibration curve for 5-year overall survival (**d**)

**Fig. 5:**
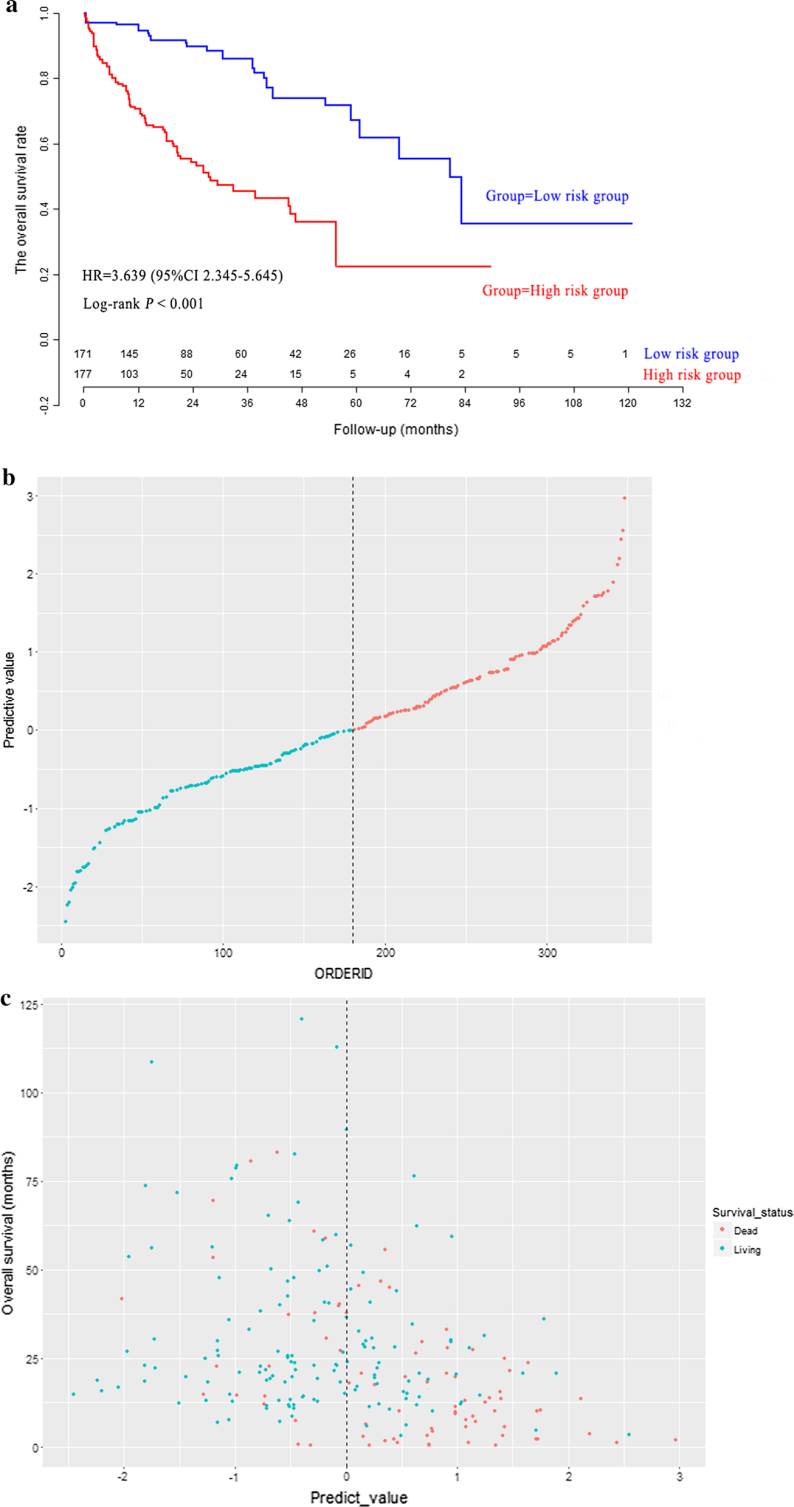
The survival curves of hepatocellular carcinoma patients in validation cohort (**a**). The distribution of LncRNA risk prediction score (**b**), survival status and survival time (**c**) in validation cohort

Time-dependent receiver operating characteristic curves were drawn to depict the clinical application of LncRNA risk prediction score for OS. The C-indexes of LncRNA risk prediction score were 0.811 (95% CI 0.769–0.853) for 1-year overall survival, 0.814 (95% CI 0.772–0.856) for 3-year overall survival and 0.796 (95% CI 0.754–0.838) for 5-year overall survival respectively (Fig. 4a). There were good agreements between predictive survival probability and actual overall survival percentage in calibration curves for 1-year survival (Fig. 4b), 3-year survival (Fig. 4c) and 5-year survival (Fig. 4d).

### Internal validation of LncRNA risk prediction score

A internal validation cohort (n = 348) was drawn by random drawing with replacement method from model cohort (n = 348). The calculating method of LncRNA risk prediction scores for patients in validation cohort was as same as the previous formula of LncRNA risk prediction score in model cohort. Then 348 HCC patients in validation cohort were stratified into low risk group (n = 174) and high risk group (n = 174) through the previous cut-off value in model cohort. The survival curve analysis (Fig. 5a) indicated that the overall survival rate in high risk group was significantly poorer than that in low risk group (*P *< 0.001). The distribution of LncRNA risk prediction score was presented in Fig. 5b. The survival status and survival time were presented in Fig. 5c. The C-index of LncRNA risk prediction score was 0.745 (95% CI 0.703–0.787) for OS in validation cohort.

### Clinical application of LncRNA risk prediction score in validation cohort

**Fig. 6:**
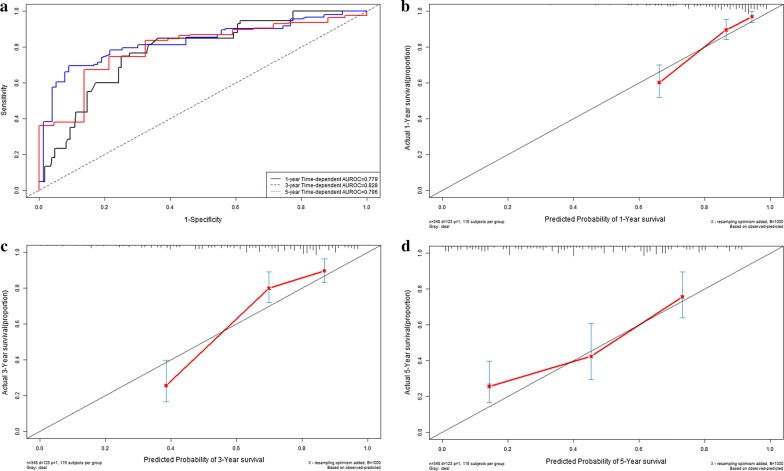
Performance of LncRNA risk prediction score in validation cohort: time-dependent receiver operating characteristic curves (**a**); calibration curve for 1-year overall survival (**b**); calibration curve for 3-year overall survival (**c**); calibration curve for 5-year overall survival (**d**)

In validation cohort, the C-indexes of LncRNA risk prediction score were 0.779 (95% CI 0.737–0.821), 0.828 (95% CI 0.786–0.870) and 0.796 (95% CI 0.754–0.838) for 1-year survival, 3-year survival and 5-year survival respectively (Fig. 6a). There were good agreements between predictive survival probability and actual overall survival percentage in calibration curves for 1-year survival (Fig. 6b), 3-year survival (Fig. 6c) and 5-year survival (Fig. 6d).

### Independence assessment of LncRNA risk prediction score

Multivariate Cox regression analyses were carried out to explore the independence of LncRNA risk prediction score for OS of HCC patients. The pathological diagnosis was carried out in accordance with the suggestions of the American Joint Committee on Cancer (AJCC). After adjusting the confounding effects of pathological parameters, gender and age, multivariate Cox regression analyses indicated that LncRNA risk prediction score was an independent influence factor for OS of HCC patients (Table 3).

**Table 3:** Univariate and multivariable Cox regression analyses

	Univariate analysis			Multivariate analysis			
	HR	95% CI	*P*-value	Coefficient	HR	95% CI	*P*-value
Model group (n = 348)							
Age (high/low)	1.254	0.887–1.772	0.199	0.204	1.227	0.838–1.797	0.294
Gender (male/female)	0.817	0.573–1.164	0.264	− 0.123	0.884	0.602–1.298	0.530
AJCC PT (T4, T3/T2, T1)	2.548	1.794–3.617	< 0.001	0.614	1.847	0.244–13.991	0.553
AJCC PN (N2, N1/N0)	1.509	1.048–2.174	0.027	− 0.132	0.876	0.542–1.417	0.590
AJCC PM (MX, M1/M0)	1.674	1.162–2.413	0.006	0.622	1.863	1.159–2.993	0.010
AJCC stage (IV, III/II, I)	2.442	1.685–3.540	< 0.001	0.169	1.184	0.158–8.868	0.870
LncRNA risk prediction score (high/low)	4.140	2.801–6.120	< 0.001	1.421	4.141	2.705–6.340	< 0.001
Validation cohort (n = 348)							
Age (year)	2.057	1.421–2.978	< 0.001	0.528	1.696	1.129–2.550	0.011
Gender (male/female)	0.777	0.542–1.113	0.169	− 0.062	0.940	0.629–1.403	0.761
AJCC PT (T4, T3/T2, T1)	1.890	1.294–2.762	0.001	0.433	1.541	0.201–11.816	0.677
AJCC PN (N2, N1/N0)	1.635	1.143–2.339	0.007	− 0.084	0.919	0.585–1.443	0.714
AJCC PM (MX, M1/M0)	1.681	1.171–2.413	0.005	0.392	1.480	0.940–2.330	0.091
AJCC stage (IV, III/II, I)	1.687	1.123–2.534	0.012	− 0.005	0.995	0.134–7.405	0.996
LncRNA risk prediction score (high/low)	3.891	2.615–5.788	< 0.001	1.292	3.639	2.345–5.645	< 0.001

The median of LncRNA risk prediction score scores was used as the cut-off values to stratify hepatocellular carcinoma patients into high risk group and low risk group

*AJCC* the American Joint Committee on Cancer, *HR* hazard ratio, *CI* confidence interval

### Survival curve analysis of ten lncRNAs in LncRNA risk prediction score

**Fig. 7:**
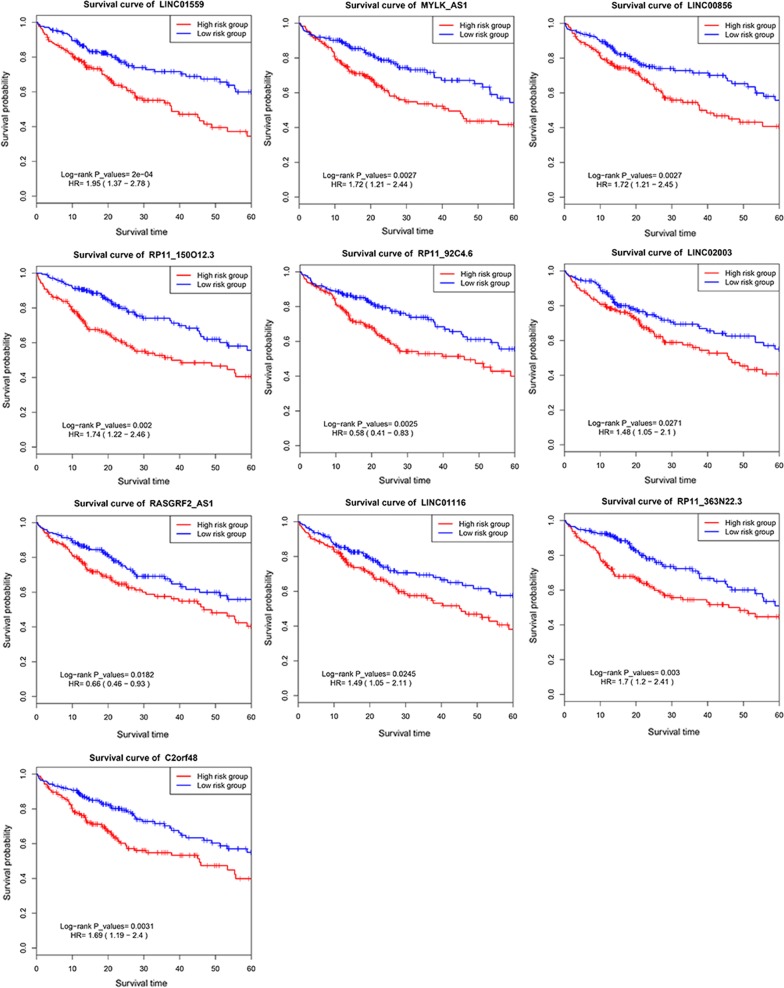
The survival curves of ten lncRNAs in LncRNA risk prediction score

The survival curve analysis of lncRNAs in LncRNA risk prediction score was present in Fig. 7. As shown in Fig. 7, OS was significantly different according to ten lncRNAs in LncRNA risk prediction score (*P *< 0.001).

### Pathological stage subgroup analysis

**Fig. 8:**
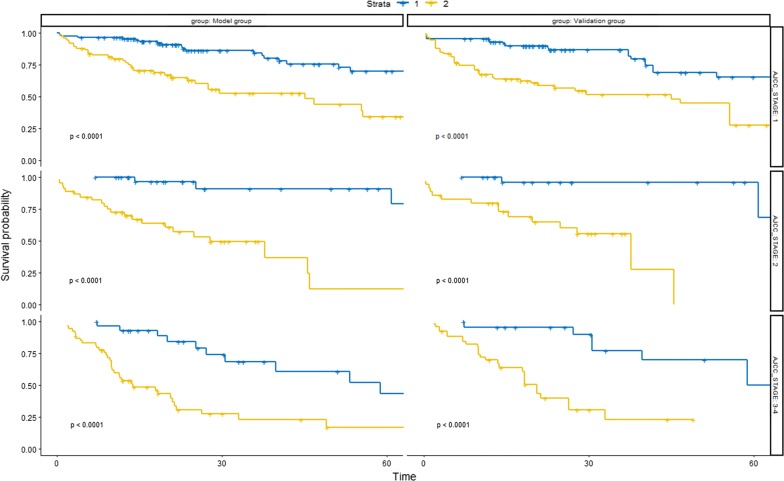
Pathological stage subgroup analysis

Pathological stage was an important influence factor for overall survival of HCC patients. As shown in Fig. 8, OS in high risk group was significantly poorer than that in low risk group in different pathological stages, indicating that the predictive performance of LncRNA risk prediction score for OS was stable and reliable in different pathological stage subgroups.

### Functional enrichment analysis

**Fig. 9:**
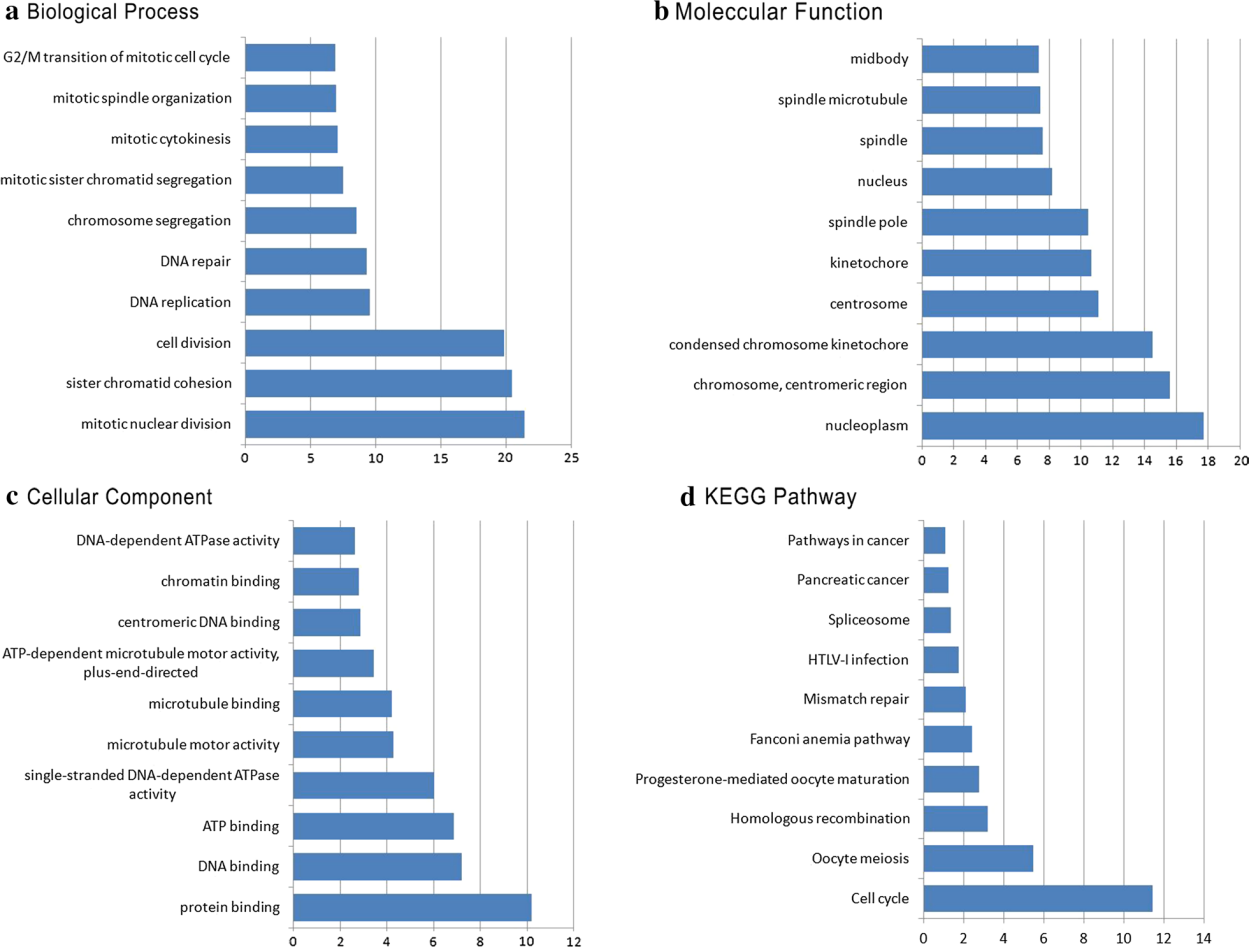
Functional enrichment analysis of prognostic signature: **a** biological process; **b** molecular function; **c** cellular component; **d** KEGG pathway. *KEGG* Kyoto Encyclopedia of Genes and Genomes

According to the criteria of *P* value < 0.05 and |Spearman correlation coefficient| > 0.7, 162 mRNA genes were significantly co-expressed with prognostic lncRNAs included in LncRNA risk prediction score. Functional enrichment analysis was performed through the Database for Annotation, Visualization and Integrated Discovery (DAVID, https://david.ncifcrf.gov/). Gene ontology (GO) biological process enrichment analysis and Kyoto Encyclopedia of Genes and Genomes (KEGG) signaling pathway analysis were presented in Fig. 9. Functional enrichment analysis indicated that the co-expressed genes were mainly enriched in mitotic nuclear division, cell division, DNA replication, DNA repair, regulation of cell cycle, DNA-dependent ATPase activity, and ATPase activity.

### Ten-group risk stratification chart

**Fig. 10:**
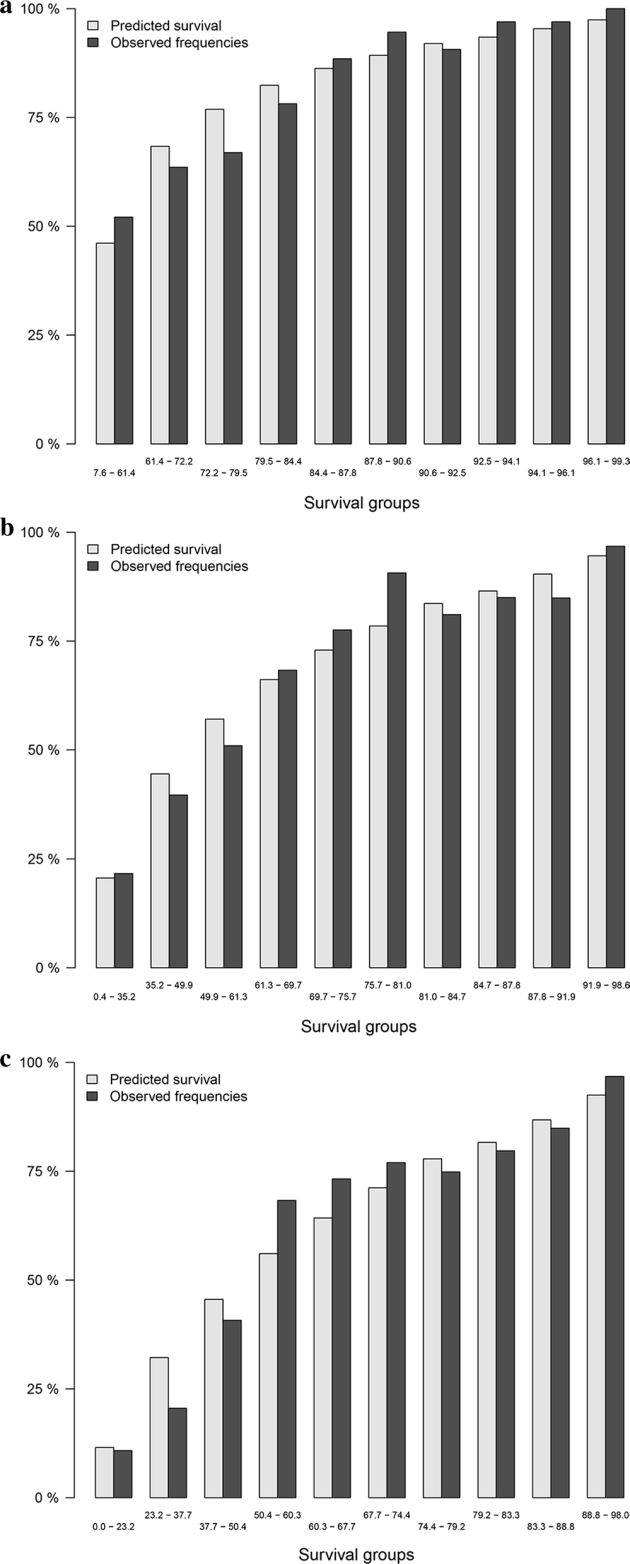
Ten-group risk stratification chart: **a** for 1-year overall survival; **b** for 2-year overall survival; **c** for 3-year overall survival

To explore the predictive performance of LncRNA risk prediction score for OS, a 10-group risk stratification chart was presented in Fig. 10 for model cohort. The discriminative ability of LncRNA risk prediction score for 1 year, 2 year, and 3 year OS were showed in Fig. 10a–c.

### Association between prognostic lncRNAs and tumors of digestive system

We further explored the association between prognostic lncRNAs and tumors of digestive system through MNDR v2.0 database (http://www.rna-society.org/mndr/index.html). MNDR v2.0 database integrated clinical evidences from 14 resources and provided a confidence score for each ncRNA-disease association.

RASGRF2_AS1, LINC00856 and LINC01116 were related with hepatocellular carcinoma (score 0.1097), stomach cancer (score 0.1097), and colorectal cancer (score 0.1097). MYLK_AS1 was related with stomach cancer (score 0.8473). RP11_150O12.3 was related with stomach cancer (score 0.4752). LINC01559 and C2orf48 were related with stomach cancer (score 0.1097).

## Discussion

The current study developed and validated a prognostic model named LncRNA risk prediction score, which was helpful to predict the individual mortality risk and identify the patients with high mortality risk. LncRNA risk prediction score could help HCC patients with high mortality risk optimize their individualized clinical decision.

LncRNA risk prediction score, as a prognostic nomogram, provided a noninvasive preoperative predictive method for overall survival of HCC patients. The nomogram predictive chart has been used as predictive tool for prediction of prognosis in different cancers [15, 16]. The present study constructed LncRNA risk prediction score for OS was based on the following points to consider: First, there is an urgent need for clinical practice to construct a preoperative predictive method to forecast the overall survival of HCC patients before further surgery. The HCC patients with high mortality risk identified by prognostic models would be more willing to accept active treatment such as surgical treatment. Second, for HCC patients without pathological diagnosis information, LncRNA risk prediction score could provide an alternative noninvasive predictive method for overall survival.

The previous prognostic models didn’t present in the current study for the following causes [8–10]. First, these prognostic models were developed based on lncRNA expression values generated on different gene detection platforms. Due to the differences between different gene detection platforms, these prognostic models couldn’t be calculated directly in the current study. Second, the previous studies further standardized the original lncRNA expression counts by using different standardization methods. The standardization methods in these previous studies reduced the repeatability and clinical applicability of these prognostic models.

The current study has the following advantages in predicting the overall survival of HCC patients: First, LncRNA risk prediction score, as a simple predictive nomogram, was easy to calculate and understand by patients. Second, the individual mortality risk was presented as percentage of mortality risk, which was easy to interpret the clinical significance of the predictive result for patients without medical knowledge. Third, since this prognostic nomogram didn’t contain pathological parameters, LncRNA risk prediction score was a noninvasive predictive method and subsequently more suitable for preoperative prediction for OS.

There were several shortcomings in the current study. First, LncRNA risk prediction score has not been validated through external study dataset. Therefore it was necessary to validate the predictive performance of LncRNA risk prediction score in different external study population. Second, the sample size of the current study was relevant small and then large prospective multicenter studies are needed to further validate the clinical value of LncRNA risk prediction score for overall survival of HCC patients. Third, the results in the present study depended on gene mining approach and lacked evidences from clinical trials. It is necessary to carry out further clinical research to verify the results in the present study.

## Conclusion

In conclusion, the current study developed and validated a prognostic model to predict the individual mortality risk of HCC patients. The LncRNA risk prediction score is helpful to identify the patients with high mortality risk and subsequently optimize the individualized treatment decision.

## Additional files

**Additional file 1.** Model cohort dataset.

**Additional file 2.** Validation cohort dataset.

**Additional file 3: Figure S1.** Heat map.

**Additional file 4: Figure S2.** Volcano map.

## Data Availability

The datasets analyzed in the current study are provided as the additional documents in the end of the current article. Smart Cancer Predictive System tools were designed by Zhiqiao Zhang from Precision Medical Development Group of Institute of Hepatology, Shunde Hospital, Southern Medical University. The following calculator is the fifth predictive tool in Smart Cancer Predictive System. The web calculator can be used by click the following URL: https://zhangzhiqiao2.shinyapps.io/Smart_cancer_predictive_system_HCC_3/.

## Abbreviations

HCC: hepatocellular carcinoma
TCGA: The Cancer Genome Atlas
GEO: the Gene Expression Omnibus
ROC: receiver operating characteristic
OS: overall survival
lncRNA: long non-coding RNA
miRNA: microRNAs
HR: hazard ratio
CI: confidence interval
AJCC: the American Joint Committee on Cancer
SD: standard deviation
DCA: decision curve analysis
ceRNA: competitive endogenous RNA
