# Supplementary figures and images for: Comprehensive bioinformatics analysis reveals potential lncRNA biomarkers for overall survival in patients with hepatocellular carcinoma: an on-line individual risk calculator based on TCGA cohort
Source: Cancer Cell Int. 2019 Jul 4;19:174. doi: 10.1186/s12935-019-0890-2 (PMC6611026; doi:10.1186/s12935-019-0890-2)

# Volcano

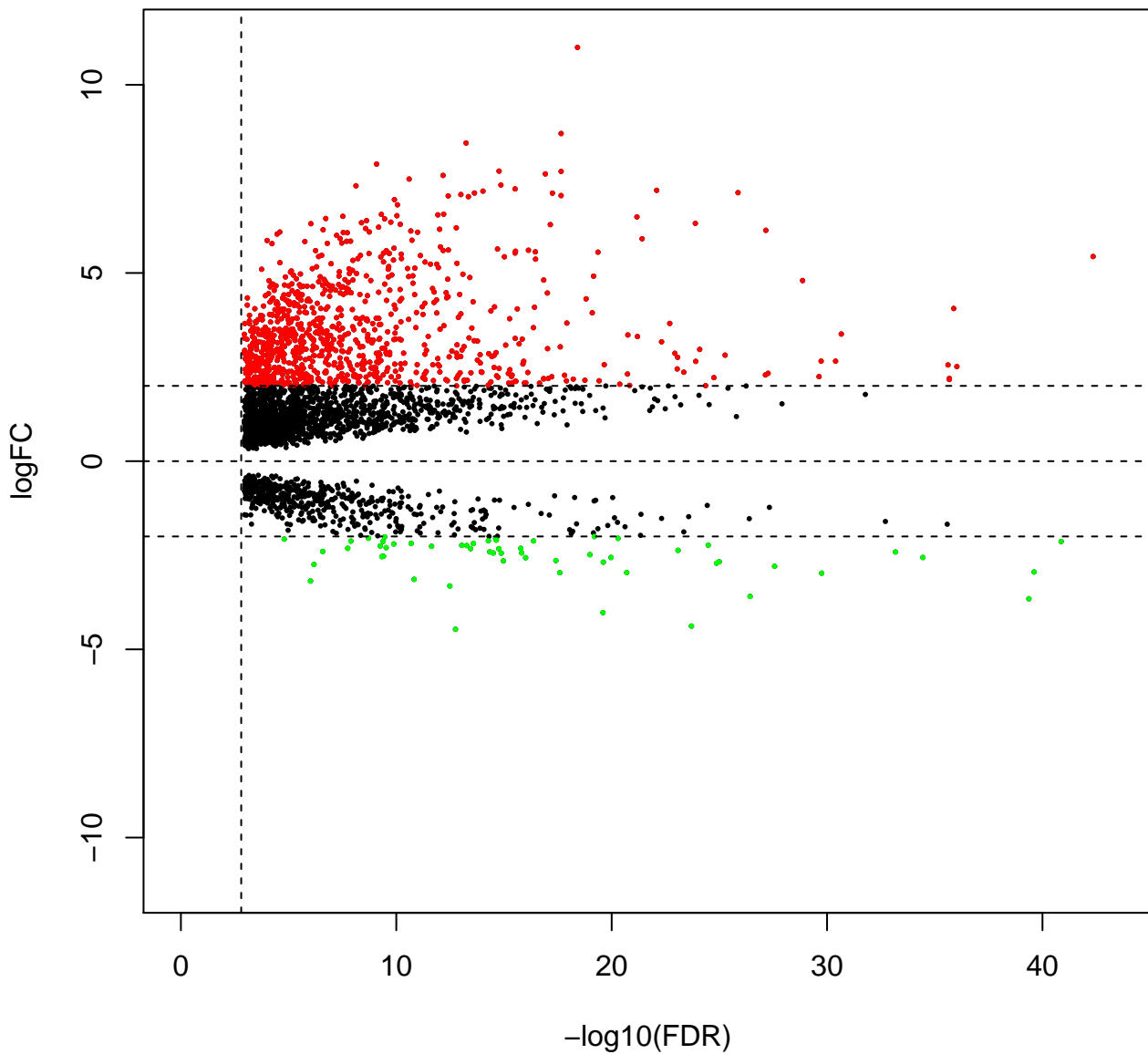

Supplement: Supplementary file 4 — Additional file 4: Figure S2. Volcano map. [file 12935_2019_890_MOESM4_ESM.pdf]
