# Supplementary material for: Comprehensive bioinformatics analysis reveals potential lncRNA biomarkers for overall survival in patients with hepatocellular carcinoma: an on-line individual risk calculator based on TCGA cohort
Source: Cancer Cell Int. 2019 Jul 4;19:174. doi: 10.1186/s12935-019-0890-2 (PMC6611026; doi:10.1186/s12935-019-0890-2)
Supplement: Supplementary file 3 — Additional file 3: Figure S1. Heat map. [file 12935_2019_890_MOESM3_ESM.pdf]

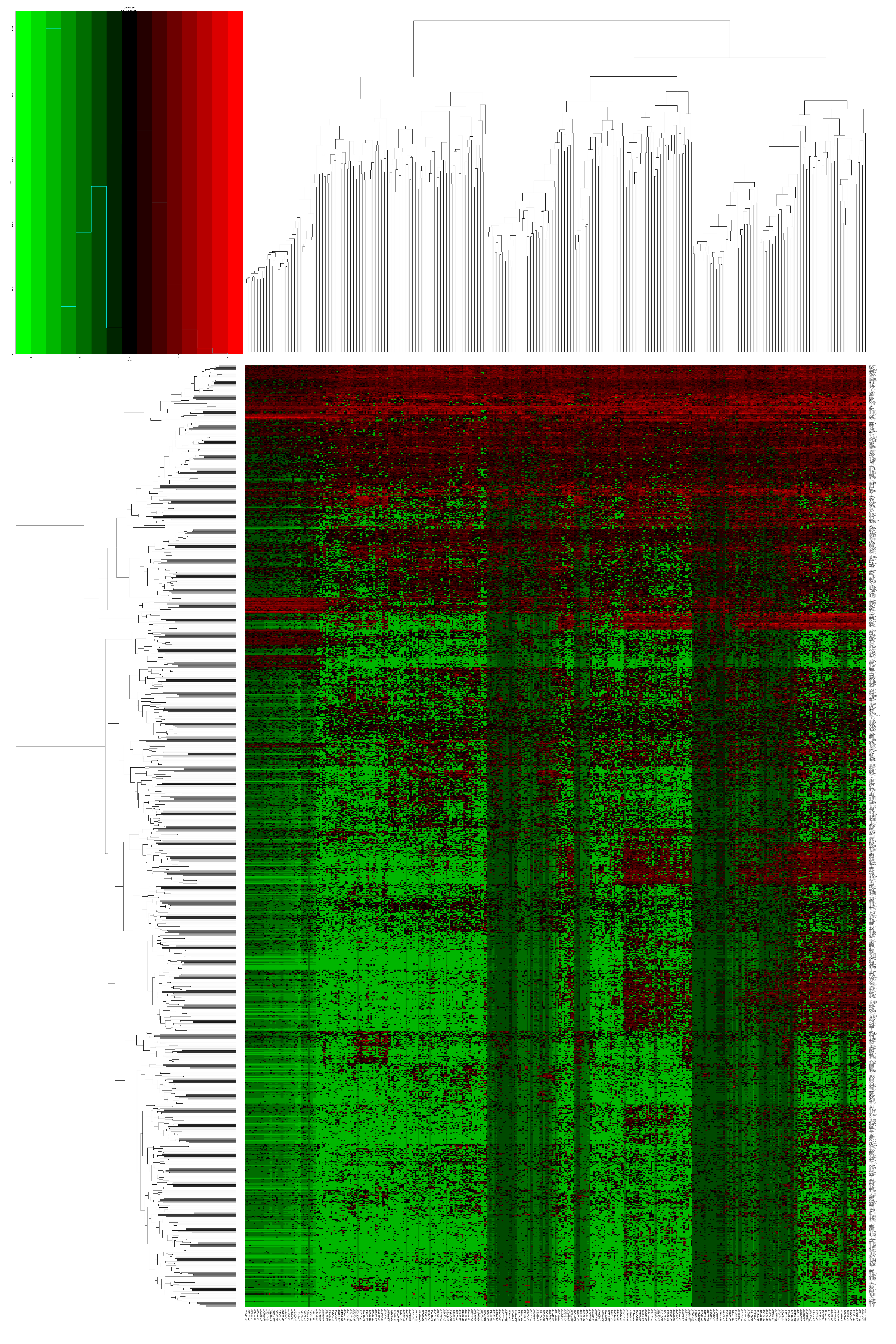

Color Key
and Histogram
1e+05
80000
60000
40000
20000
0
Count
−4
−2
0
2
4
Value
